# Dynamic contrast-enhanced magnetic resonance imaging in paediatric brain tumours systematically reviewed

**DOI:** 10.1007/s00247-026-06600-7

**Published:** 2026-04-01

**Authors:** Nathalie Ringrose, Şeyma Atmaca, Vera C. Keil, Yeva Prysiazhniuk

**Affiliations:** 1 Department of Radiology and Nuclear Medicine, Amsterdam UMC, De Boelelaan 1117, Amsterdam, 1081HV Netherlands; 2 Faculty of Medicine, Vrije Universiteit Amsterdam, Amsterdam, Netherlands; 3 Imaging and Biomarkers, Cancer Center Amsterdam, Amsterdam, Netherlands; 4 Second Faculty of Medicine, Department of Pathophysiology, Motol and Homolka University Hospital, Prague, Czech Republic

**Keywords:** Brain tumour, Dynamic contrast-enhanced magnetic resonance imaging, Glioma, Paediatrics

## Abstract

**Background:**

Dynamic contrast-enhanced magnetic resonance imaging (DCE MRI) is an advanced imaging technique that quantifies blood-brain permeability using dynamic contrast uptake.

**Objective:**

The clinical utility of DCE in paediatric brain tumours is unclear. This systematic review evaluates the efficacy of DCE for tumour differentiation and progression assessment in paediatric brain tumours, and summarises current technical implementation to inform clinical practice.

**Materials and methods:**

A string-based literature search was performed in PubMed and Web of Science. Original articles evaluating the utility of DCE were included. A modified quality assessment of diagnostic accuracy studies-2 (QUADAS-2) instrument evaluated the risk of bias.

**Results:**

Nine studies (2008-2025) were eligible (sample size 6-72 cases). Six studies investigated low-grade versus high-grade differentiation in mixed paediatric tumours (cumulative sample *n*=196) with successful discrimination through *K*^trans^ and/or *k*_ep_ in three studies (60 patients). Discrimination of two distinct histologies was usually more successful. Two studies evaluated the response to different treatments. Results for survival prediction based on DCE parameters were not promising. One study attempted to predict tumour aggressiveness in optic pathway glioma with good prognostic capacity for *K*^trans^. DCE technical execution varied substantially among studies and was usually not compliant with current guidelines. Meta-analyses were impossible.

**Conclusion:**

DCE may be of added value to discriminate between two different paediatric brain tumour entities, but a general discrimination potential between low- and high-grade lesions is doubtful. More studies and greater technical homogeneity are needed to investigate the technique’s prognostic potential for paediatric cohorts.

**Graphical abstract:**

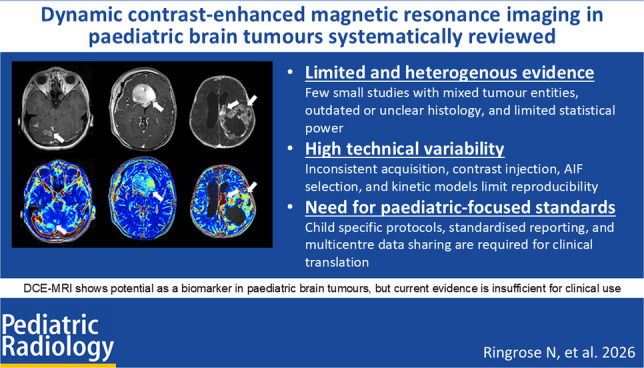

**Supplementary Information:**

The online version contains supplementary material available at 10.1007/s00247-026-06600-7.

## Introduction

Brain tumours rank as the most lethal, as well as the second most common type of cancer in paediatric patients [1]. Paediatric patients are in need of biomarkers that avoid surgery to classify brain tumours and evaluate treatment response due to their vulnerability and the frequently surgically unfavourable tumour locations. Reliable noninvasive biomarkers may also improve cognitive outcome in this population [2].

Magnetic resonance imaging (MRI) is the key pillar of brain tumour therapy planning and surveillance, including in the paediatric population. Still, conventional MRI tumour protocols often fail to adequately capture the biological complexity of brain tumours, limiting their ability to accurately assess tumour vitality and malignancy. Advanced MRI techniques, on the other hand, show potential in improving the diagnostic performance in differentiating brain tumours in the paediatric population as well as the evaluation of treatment response [3].

**Fig. 1:**
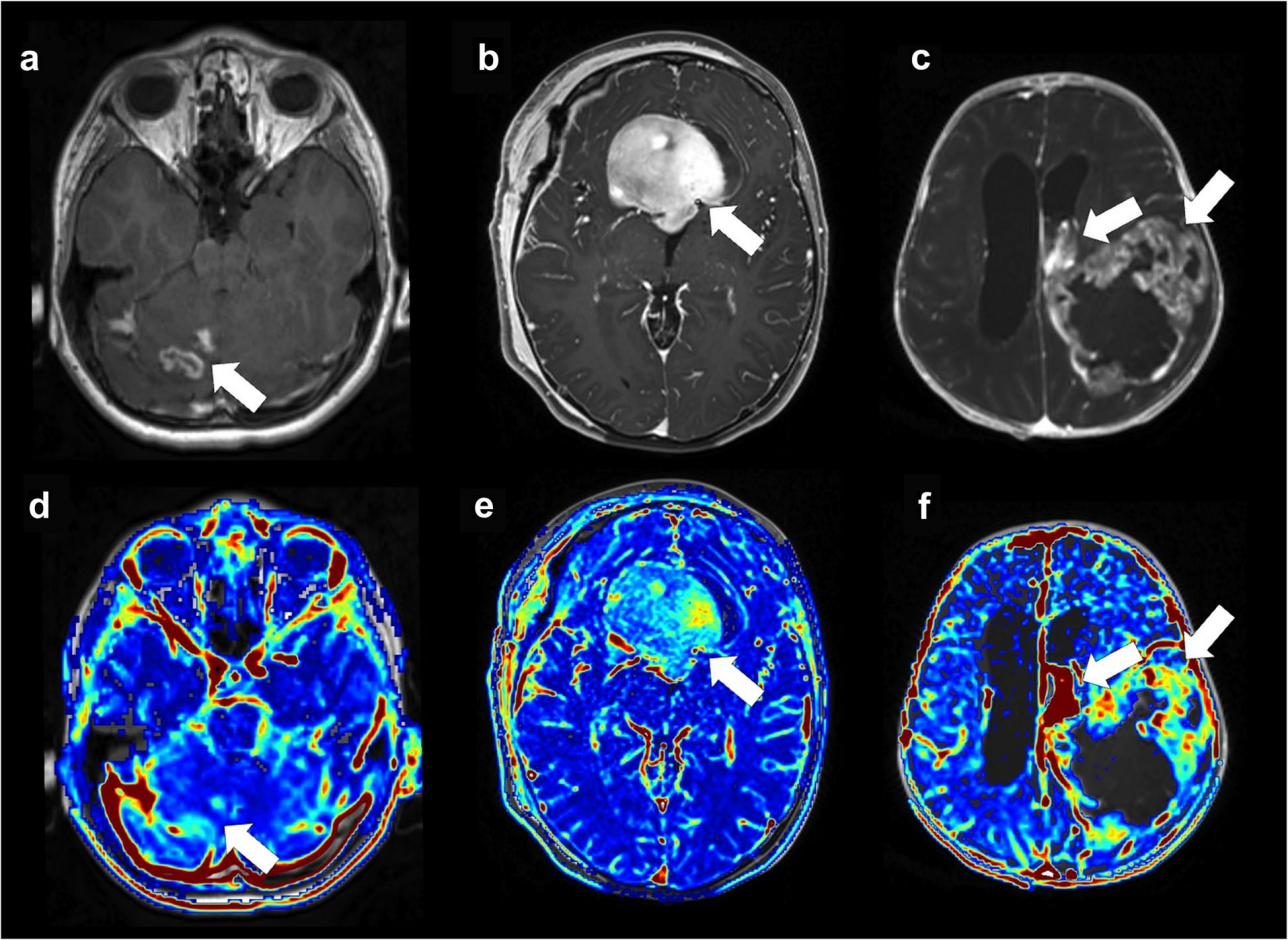
Axial post-contrast T1-weighted structural (**a**-**c**) and dynamic contrast-enhanced (**d-f**) magnetic resonance images show brain tumours in three children. **a**,** d** A 12-year-old boy with a rosette-forming glioneuronal tumour (World Health Organization (WHO) grade 1). **b**,** e** A 5-year-old girl with pilocytic astrocytoma, *KIAA1549::BRAF* fusion-positive (WHO grade 1). **c**, **f** A 1-month-old girl with a supratentorial ependymoma, *YAP1*-fusion-positive (WHO grade 3)

Dynamic contrast-enhanced (DCE) MRI is an advanced quantitative MRI technique that is well-established for evaluating adult-type brain tumours [4]. DCE is a contrast agent injection-dependent sequence aiming to deliver insights into blood-brain barrier integrity by quantifying vascular permeability and perfusion-related parameters [5]. A significant correlation has been observed between DCE kinetic parameters and tumour grade in adult brain tumours [6]. However, the most common low-grade paediatric brain tumour, pilocytic astrocytoma, also typically shows blood-brain barrier disruption with pronounced contrast enhancement, in contrast to the more restrained enhancement patterns observed in adult low-grade gliomas or other paediatric low-grade tumours (Fig. 1). Nevertheless, systematic data on DCE measurements in paediatric gliomas remain scarce and underrepresented.

From a technical perspective, the Radiological Society of North America’s Quantitative Imaging Biomarkers Alliance (QIBA) suggested standardised scan parameters and processing for adults to increase DCE reproducibility without a paediatric counterpart [7]. Children differ, however, from adults in several aspects relevant to DCE measurements, including brain volume, cardiac output, cerebral blood flow, haematocrit, and contrast agent response, as well as in practical implementation factors such as cannula size and achievable injection rates [8].

A structured evaluation of DCE for paediatric brain tumour patients is therefore imperative. This systematic literature review was conducted to (1) evaluate the value of DCE in the diagnostic assessment of paediatric brain tumours and their therapy and (2) deliver an overview of DCE paediatric scan protocols for the radiological community.

## Methods

### Protocol

The literature search strategy, including the construction of the search string, was developed in accordance with the Population, Intervention, Comparator, Outcome framework (PICO; Table 1). The population comprised paediatric patients, including infants and children. The intervention of interest was DCE MRI. No explicit comparator was specified, as the primary aim of this review was to comprehensively characterise the application of DCE, including all feasible (semi-)quantitative parameters, rather than to assess comparative diagnostic performance. The outcome was the evaluation of brain tumours. For each PICO component, a comprehensive set of keywords and synonyms was defined and combined using the building block approach.

**Table 1:** Search string for Web of Science and PubMed

Component	Search terms
Population: paediatric	Paediatrics, Infant, Child, Paediatric, Infant, Child, Children, Children’s, Childhood
Intervention: dynamic contrast-enhanced magnetic resonance imaging	Dynamic contrast-enhanced Magnetic Resonance Imaging, Dynamic contrast-enhanced MRI, Dynamic contrast-enhanced Imaging, Dynamic contrast-enhanced Image, Dynamic CE MRI, Dynamic CE Magnetic Resonance Imaging, Dynamic CE Imaging, Dynamic CE Image, DCE MRI, DCE Magnetic Resonance Imaging, DCE Magnetic Resonance Image, DCE Imaging, DCE Image, Dynamic contrast-enhanced Perfusion, DCE Perfusion, DCE Perfusion MRI, DCE Perfusion Magnetic Resonance Imaging, DCE Perfusion Magnetic Resonance Image, Dynamic contrast-enhanced Perfusion MRI, Dynamic contrast-enhanced Perfusion Magnetic Resonance Imaging, DCE Perfusion Imaging, DCE Perfusion Image, Dynamic contrast-enhanced T1-Weighted MRI, DCE T1-Weighted MRI, Dynamic contrast-enhanced T1-Weighted Magnetic Resonance Imaging, DCE T1-Weighted Magnetic Resonance Imaging, Dynamic contrast-enhanced T1-Weighted Magnetic Resonance Image, DCE T1-Weighted Magnetic Resonance Image, Dynamic contrast-enhanced T1-Weighted Imaging, DCE T1-Weighted Imaging, Dynamic contrast-enhanced T1-Weighted Image, DCE T1-Weighted Image, Dynamic contrast-enhanced Perfusion T1-Weighted Magnetic Resonance Imaging, T1-Weighted Dynamic Contrast-Enhanced Brain MRI, T1-Weighted Dynamic Contrast-Enhanced Brain Magnetic Resonance Imaging, T1-Weighted Dynamic Contrast-Enhanced Brain Magnetic Resonance Image, T1-Weighted Dynamic contrast-enhanced Magnetic Resonance Imaging, T1-Weighted Dynamic Contrast-Enhanced MRI, T1-Weighted Dynamic Contrast-Enhanced Imaging
Outcome: brain tumour	Brain Neoplasms, Brain Stem Neoplasms, Brain Tumour, Brain Tumour, Brian Cancer, Brian Carcinoma, Brain Stem Tumour, Brain Stem Tumour, Cerebral Tumour, Cerebral Neoplasm, Cerebral Cancer, Cerebral Carcinoma, Cerebellar Neoplasms, White Matter Neoplasm, White Matter Tumour, White Matter Tumour, Grey Matter Neoplasm, Grey Matter Tumour, Grey Matter Tumour, Brain Metastasis, Diffuse Low Grade Tumour, Diffuse Low Grade Tumour, Diffuse Low Grade Neoplasm, Low Grade Tumour, Low Grade Tumour, Low Grade Neoplasm, Diffuse High Grade Tumour, Diffuse High Grade Tumour, Diffuse High Grade Neoplasm, High Grade Tumour, High Grade Tumour, High Grade Neoplasm, Subependymal Tumour, Subependymal Tumour, Subependymal Neoplasm, Glioneuronal Tumour, Glioneuronal Tumour, Glioneuronal Neoplasm, Neuronal Tumour, Neuronal Tumour, Neuronal Neoplasm, Dysembryoplastic Neuroepithelial Tumour, Dysembryoplastic Neuroepithelial Tumour, Dysembryoplastic Neuroepithelial Neoplasm, Neuroepithelial Tumour, Neuroepithelial Tumour, Neuroepithelial Neoplasm, Ependymal Tumour, Ependymal Tumour, Ependymal Neoplasm, Choroid Tumour, Choroid Tumour, Choroid Neoplasm, Choroid Plexus Tumour, Choroid Plexus Tumour, Choroid Plexus Neoplasm, Choroid Plexus Neoplasms, Choroid Plexus Carcinoma, Teratoid Tumour, Teratoid Tumour, Rhabdoid Tumour, Rhabdoid Tumour, Mesenchymal Tumour, Mesenchymal Tumour, Mesenchymal Neoplasm, Non-Meningothelial Tumour, Non-Meningothelial Tumour, Non-Meningothelial Neoplasm, Fibroblastic Tumour, Fibroblastic Tumour, Fibroblastic Neoplasm, Myofibroblastic Tumour, Myofibroblastic Tumour, Myofibroblastic Neoplasm, Primary Intracranial Sarcoma, Chondrogenic Tumour, Chondrogenic Tumour, Chondrogenic Neoplasm, Notochordal Tumour, Notochordal Tumour, Notochordal Neoplasm, Intracranial Tumour, Intracranial Tumour, Intracranial Neoplasm, Infratentorial Neoplasms, Supratentorial Neoplasms, Hypothalamic Neoplasms, Glioma, Subependymal, Gliosarcoma, Cerebral Ventricle Neoplasm, Infratentorial Neoplasm, Supratentorial Neoplasm, Hypothalamic Neoplasm, Diffuse Intrinsic Pontine Glioma, Glioma, Glioma, Diffuse Low Grade Glioma, Low Grade Glioma, Diffuse High Grade Glioma, High Grade Glioma, Diffuse Midline Glioma, Midline Glioma, Diffuse Hemispheric Glioma, Hemispheric Glioma, Diffuse Paediatric High Grade Glioma, Infant Type Hemispheric Glioma, Circumscribed Astrocytic Glioma, Astrocytic Glioma, Choroid Glioma, Astrocytoma, Astrocytoma, Diffuse Astrocytoma, Pilocytic Astrocytoma, High Grade Astrocytoma, Pleomorphic Xanthoastrocytoma, Xanthoastrocytoma, Subependymal Giant Cell Astrocytoma, Giant Cell Astrocytoma, Desmoplastic Infantile Astrocytoma, Oligodendroglioma, Oligodendroglioma, Oligodendroblastoma, Oligodendroglioma, Glioblastoma, Glioblastoma, Neoplasms, Neuroepithelial, Polymorphous Low-Grade Neuroepithelial Tumour, Polymorphous Low-Grade Neuroepithelial Neoplasm, Neuroepithelial Tumour, Neuroepithelial Neoplasm, Ganglioglioma, Desmoplastic Infantile Ganglioglioma, Ganglioglioma, Angiocentric Glioma, Astroblastoma, Neurocytoma, Neurocytoma, Liponeurocytoma, Ependymoma, Subependymoma, Ependymoma, Ependymoma, Medulloblastoma, Medulloblastoma, Neuroblastoma, Neuroblastoma, Pineocytoma, Pinealoma, Pineoblastoma, Schwannoma, Neurofibroma, Perineurioma, Paraganglioma, Meningioma, Hemangioblastoma, Medullary Hemangioblastoma, Chondrosarcoma, Mesenchymal, Mesenchymal Chondrosarcoma, Craniopharyngioma, Craniopharyngioma, Pituitary Neoplasm, Pituitary Adenoma, Pituitary Blastoma

Institutional review board (IRB) approval was waived for this research. The search strategy and corresponding search strings were developed by x.x. and x.x. and independently reviewed and validated by Y.P. In stage 1, only titles and abstracts were independently assessed by N.R. and S.A.; no discrepancies were identified. In stage 2, full-text articles were independently reviewed by all authors, with final inclusion decisions reached by consensus. An additional hand search was conducted, thoroughly examining references cited in the selected articles to identify additional literature relevant to the research question. No lower publication year threshold was applied. The upper year threshold was implicitly defined by the date of the literature search. A detailed description of the search, retrieval and selection is provided in Supplementary Material 1. Modified quality assessment of diagnostic accuracy studies-2 (QUADAS-2) risk of bias assessments was performed by all authors, with at least two raters evaluating each article [9]. Disagreements were resolved by consensus. Compliance with QIBA recommendations for DCE implementation and reporting was assessed for each selected study by an MRI physicist with 4 years of clinical neuroimaging experience (Y.P.).

### Search strategy

The search was carried out on the PubMed and Web of Science databases on August 31, 2025. The search strings were constructed using the predefined PICO components and their associated keywords and synonyms, combined using Boolean operators according to the building block method.

### Eligibility assessment

Publications that included both adult and paediatric patients were needed to allow for the extraction of results for paediatric patients only. All publications were evaluated for putative cohort overlap, with suggestive criteria including similar patient populations reported by the same research group across multiple publications, originating from the same institution, and using identical acquisition protocols. When counting the total number of cases studied with DCE for any kind of research question, we therefore only counted the larger sample of one kind of tumour. This was not done if the research question was the focus and research questions differed between the studies. The diagnosis of a paediatric-type brain tumour was ideally based upon recent pathology classification systems and tissue. Still, tumours without histopathological verification of diagnosis were not excluded, as paediatric tumour patients sometimes do not undergo tissue-based tumour verification, e.g. due to a challenging tumour location. Only English-language publications were included. Case series of five or more patients were allowed due to the low incidence of some paediatric brain tumours.

The exclusion criteria comprised studies that did not involve paediatric patients and articles that did not specifically address the use of DCE as a diagnostic tool. Publications not containing original data were also excluded, as were case reports with fewer than five cases. These criteria were used during search stages 1 and 2.

### Data extraction and risk of bias assessment

Data extraction included study design, tumour and patient characteristics (number of patients, sex, age, tumour grade and type), and main findings measured in parameters including transfer constant from plasma into the extravascular extracellular space (*K*^trans^) and back (*k*_ep_), extravascular extracellular space volume per unit tissue volume (*v*_e_), and blood plasma volume fraction (*v*_p_). Study results based on pharmacokinetic modelling techniques, including dual-compartment modelling and deconvolution analysis to derive blood volume and flow from DCE data to provide complementary insights into perfusion, were not included. A modified QUADAS-2 instrument was applied (Supplement Material 2) [9]. Eligibility for meta-analysis was considered based on the homogeneity of multiple aspects, including imaging protocol, tumour type, and treatment status. The availability of ≥5 studies with most QUADAS-2 categories scoring low or medium risk of bias was the liberal minimum for a meta-analysis. Otherwise, a narrative synthesis summarised the findings.

## Results

### Overview

**Fig. 2:**
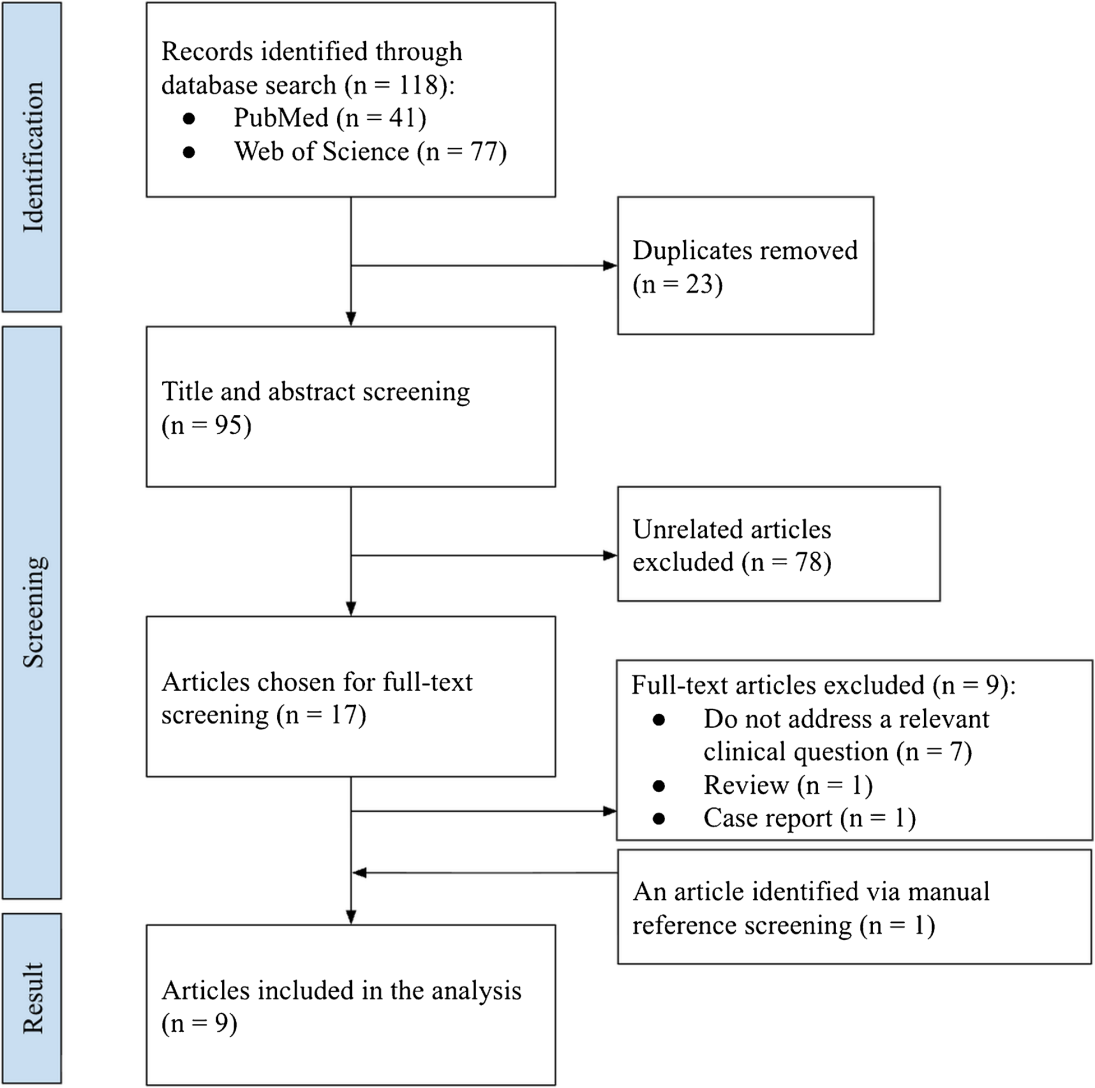
Flow chart of the study selection process

Figure 2 illustrates the retrieval. The searches rendered 118 articles. After duplicate removal, 95 articles remained for stage 1 screening. Seventy-eight articles not meeting the criteria were excluded. Seventeen articles remained for stage 2. At the end of the screening process, eight articles matched the inclusion and exclusion criteria [10–17]. Hand searching resulted in one additional relevant article [18].

### Study characteristics and summary of the main study findings

Table 2 presents the study characteristics, including study design, study population, and main results related to the research question. Technical DCE sequence details are provided in Table 3.

**Table 2:** Study characteristics and results

No.	Author	Study design	Study population	Main results
1	Arevalo-Perez J, 2024 [17]	Retrospective	6 patients (6 histologically confirmed), 3 girls, 6–16 years old; 2 low-grade ependymoma, 4 anaplastic ependymoma	Relative *v*_p, max_ shows potential for differentiating between low-grade and high-grade ependymomas; however, the study population was too small to draw definitive conclusions
2	Gupta PK, 2017 [15]	Retrospective	64 patients (64 histologically confirmed), 8 months-18 years old; 44 high-grade tumours (incl. glioblastomas, anaplastic astrocytomas, anaplastic ependymomas, medulloblastomas, choroid plexus carcinoma, atypical teratoid rhabdoid tumours, gangliogliomas) and 20 low-grade tumours (incl. astrocytomas, ganglioglioma, subependymal giant cell astrocytoma, dysembryoplastic neuroepithelial tumours, pilocytic astrocytomas)	Relative blood volume and *v*_p_ were significantly different between high- and low-grade tumours. *v*_p_ differentiated low-grade tumours from high-grade with sensitivity 0.75 and specificity 0.65 (cutoff 0.0135). *k*_ep_ demonstrated a significant difference between posterior fossa ependymomas and medulloblastomas, whereas *v*_e_, differentiated not only posterior fossa ependymomas from medulloblastomas but also pilocytic astrocytomas from medulloblastomas
3	Ho CY, 2025 [16]	Retrospective	72 patients (72 histologically confirmed), 44 boys, 1.7–211.7 months old; 36 high-grade tumours (incl. medulloblastomas, atypical teratoid/rhabdoid tumours, anaplastic ependymomas, diffuse midline gliomas, CNS embryonal tumours NOS, high-grade gliomas, anaplastic astrocytomas, ganglioblastoma) and 36 low-grade tumours (incl. pilocytic astrocytomas, dysembryoplastic neuroepithelial tumours, gangliogliomas, desmoplastic infantile ganglioglioma, diffuse astrocytoma, ependymoma, low-grade astrocytoma, low-grade glial neoplasm, low-grade neuroepithelial neoplasm, optic chiasm glioma, pilomyxoid astrocytoma, pleomorphic xanthoastrocytoma)	None of the DCE parameters showed a significant difference between low- and high-grade gliomas after statistical correction
4	Jost SC, 2008 [14]	Retrospective	27 patients with optic pathway gliomas (unclear histology status); 14 had OPGs associated with neurofibromatosis type 1, 13 had sporadic OPGs; 11 were classified as “clinically stable”, 16 as “clinically aggressive”	“Clinically aggressive” OPGs demonstrated significantly higher *K*^PS^ compared with “clinically stable” OPGs. Among sporadic cases, tumours classified as “clinically aggressive” also exhibited significantly greater permeability values than their “clinically stable” counterparts
5	Rochetams BB, 2017 [13]	Prospective	18 patients (18 histologically confirmed), 9 girls, 0.47–15.92.47.92 years old; 6 patients with high-grade tumours (incl. rhabdoid tumour, medulloblastomas, high-grade gliomas) and 4 low-grade tumours (pilocytic astrocytomas, DNET, low-grade glioma)	Types of concentration-time curves are presented for brain tumours. There was a significant difference in *K*^trans^ between grade IV and I tumours, but there was no difference in *K*_ep_ and *v*_e_. *K*^trans^ and *v*_e_ (but not *K*_ep_) were significantly different in tumour when compared to non-pathological surrounding tissue
6	Vajapeyam S, 2020 [12]	Prospective	53 patients (histology likely present considering the other two studies), 33 girls, 2.5–12.9.5.9 years, 3 excluded from the analysis; all 50 patients had DIPGs, 43 died, 45 experienced a PFS event	Higher mean *K*^trans^ and mean *v*_e_ were associated with shorter OS and PFS. Maximum *K*^trans^ was associated with PFS
7	Vajapeyam S, 2018 [11]*	Retrospective	41 patients (40 histologically confirmed), 23 boys, 0.3–16.76.3.76 years old; 31 infratentorial and 10 supratentorial tumours; 16 low-grade (7 pilocytic astrocytomas, 5 low-grade gliomas, 1 mature teratoma, 1 atypical meningioma, 1 low-grade ganglioglioma, and 1 low-grade mixed germ cell tumour), 25 high-grade tumours (12 medulloblastomas, 4 glioblastomas, 4 anaplastic ependymomas, and 1 each of atypical teratoid/rhabdoid tumour, embryonal tumour not otherwise specified, choroid plexus carcinoma, embryonal tumour with rhabdoid features, and diffuse midline glioma)	*K*^trans^, *k*_ep_, *v*_e_ showed a significant difference between high- and low-grade tumours. ROC analysis has demonstrated good discriminatory performance for *K*^trans^ (AROC=0.883, CI 0.781–0.984.781.984), *k*_ep_ (AROC=0.908, CI 0.815–1.0.815.0), and *v*_e_ (AROC=0.843, CI 0.713–0.972.713.972) in distinguishing between high- and low-grade tumours
8	Vajapeyam S, 2017 [10]*	Retrospective	38 patients (38 histologically confirmed), 24 boys, 0.3–18.14.3.14 years; 18 low-grade tumours (7 pilocytic astrocytomas, 3 low-grade gliomas with piloid features, 3 low-grade gliomas, 1 low-grade ependymoma, 1 atypical meningioma WHO II, 1 hemangioblastoma grade I, 1 ganglioglioma grade I–II, 1 low-grade histiocytic sarcoma) and 20 high-grade tumours (11 medulloblastomas, 3 glioblastoma multiformes, 2 anaplastic ependymomas, 1 high-grade sarcoma, 1 choroid plexus carcinoma, 1 germinomatous germ cell tumour, and 1 high-grade glioma)	*K*^trans^, *k*_ep_, *v*_e_ showed a significant difference between populations of high- and low-grade tumours with high sensitivity (>0.7) and specificity (>0.82)
9	Zukotynski KA, 2013 [18]	Prospective	24 patients, 13 girls; 7 supratentorial HGGs, 9 LGGs, 4 BSGs, 2 medulloblastomas, 2 ependymomas; however, 21 patients with *K*_ps, max_ measurements (unclear histology status)	No statistically significant difference in *K*_ps, max_ was observed between children with HGG/BSG and those with LGG. *K*_ps, max_ was not significantly correlated with PFS

*AROC*, area under the receiver operating curve; *BSG*, brainstem glioma; *CNS*, central nervous system; *DIPG*, diffuse intrinsic pontine glioma; *DNET*, dysembryoplastic neuroepithelial tumour; *HGG*, high-grade glioma; *LGG*, low-grade glioma; *NOS*, not otherwise specified; *OPG*, optic pathway glioma; *OS*, overall survival; *PFS*, progression-free survival; *WHO*, World Health Organization

*Vajapeyam et al. [11] and Vajapeyam et al. [10] were considered to contain cohort overlap. This included all the patients from the 2018 study, as well as 1 low-grade ependymoma, 1 hemangioblastoma grade I, 1 low-grade histiocytic sarcoma, and 1 high-grade sarcoma included in Vajapeyam et al. [10]. Based on diagnostic composition, the potentially overlapping cohorts comprised 14 low-grade gliomas (LGG), accounting for 87.5% of the LGG cases reported in Vajapeyam et al. [11] and 77.7% in Vajapeyam et al. [10], as well as 18 high-grade gliomas (HGG), representing 72% and 90% of the HGG cohorts in the 2018 and 2017 studies, respectively

**Table 3:** Technical details of dynamic contrast-enhanced magnetic resonance imaging sequence

No.	Author	Scanner and field strength	Head coil type	Contrast agent type and dose	Sequence type and flip angle (FA)	Number of phases/time resolution	Coverage/in-plane sampling (FOV/matrix size)	T1/B1 map acquisition	Processing
1	Arevalo-Perez J, 2024 [17]	Mixed: 1.5-T Optima GE, 3-T Signa Premier GE	8-channel head coil	Gatobutrol (Gadavist, Bayer) 0.1 mmol/kg, delivered with a power injector at 2–3 mL/s via an 18–21-gauge venous catheter	3D T1-weighted fast-spoiled (fast echo-spoiled) gradient-echo sequence; FA 25°; TR 4–5 ms; TE 1–2 ms	Temporal resolution 5–6 s; 10 pre-injection phases and 30 post-injection phases	FOV 24 cm, matrix 128×128, slice thickness 3 mm, 10–12 axial images	No T1/B1 mapping reported	Software: NordicIce Preprocessing: spatial and temporal smoothing AIF: individually computed from MCA Model: extended Tofts two-compartment
2	Gupta PK, 2017 [15]	3-T Philips	15-channel head coil	Gd-BOPTA (Multihance, Bracco, Italy) 0.1 mmol/kg, injected via a power injector at 1.5–2 mL/s via 22–24-gauge cannulas	T1 fast gradient echo; FA 10°; TR/TE=5.0/1.4 ms	Temporal resolution ~3.9 s, 32 dynamics (4 pre-injection)	FOV 24 cm, 128×128, slice thickness 6 mm, 12 axial slices	Pre-contrast T1 mapping reported	Software: not specified Preprocessing: not specified AIF: automated extraction Model: leaky tracer kinetic model
3	Ho CY, 2025 [16]	3-T Magnetom Skyra Siemens	Not specified	Gd-BOPTA, two doses 0.1 mmol/kg each, injected via power injector at 5 mL/s when 18–20 G IV access was possible; 24 G used in smaller children	T1-weighted gradient echo; FA 10°; TR/TE=1.54/3.91 ms	100 time points over 4.5 min, ~2.7 s per phase; unclear how many phases before/after injection	FOV not reported, matrix 154×192, slice thickness 5 mm, 20 axial slices	No T1/B1 mapping reported	Software: IDL package “Qimage” Preprocessing: FSL for motion correction and registration AIF: Manual MCA ROI Model: extended Tofts model
4	Jost SC, 2008 [14]	Mixed: 1.5-T Sonata Siemens and 3-T Trio Siemens	Not specified	Contrast agent not specified, 0.1 mmol/kg, injection method not specified	T1-weighted 3D FLASH, FA not specified, TR/TE=30/6 ms	Dynamic series not stated, dynamic duration 6 min	FOV ~128×128 mm^2^, matrix 128×128, in-plane voxel 1 mm^2^, slice thickness 3, 16 axial slices	VFA T1 mapping (FAs 10°, 15°, 25°)	Software: customised MATLAB Preprocessing: coregistration with Intelli-link AIF: blood ROI selection not specified Model: Patlak
5	Rochetams BB, 2017 [13]	1.5-T Magnetom Aero Siemens	20-channel head coil	Gadoteric acid (Dotarem, Guerbet), 0.2 mL/kg, injected with power injector at 1 mL/s via peripheral 22G IV	3D T1-weighted gradient echo; FA 12°; TR/TE=4.46/1.72 ms	56 time points, temporal resolution ~3.1 s, dynamic duration 2:53 min	FOV 230×186 mm^2^, matrix 166×256, slice thickness 3 mm	No T1/B1 mapping reported	Software: from vendor (Syngo MR Tissue 4D, Siemens) Preprocessing: not specified AIF: automatic extraction Model: extended Tofts two-compartment
6	Vajapeyam S, 2020 [12]	3 T (unspecified)	Not specified	Gadobutrol, two half-doses (0.05 mmol/kg each), injection not specified	3D T1-weighted fast gradient echo; FA 15°; TR 4 s, TE minimum	50 timepoints, ~7 s/phase, contrast injection after 20 s	FOV 24 cm, matrix not specified, slice thickness 5 mm	VFA T1 mapping (FAs 15°, 10°, 5°, 2°)	Software: DynaCAD (Invivo) with OmniLook Preprocessing: not specified AIF: not specified Model: extended Tofts two-compartment
7	Vajapeyam S, 2018 [11]	3-T Siemens	Not specified	Gadobutrol, 0.1 mL/kg at injection rate 2 mL/s, the rest not specified	3D T1-weighted fast gradient echo; FA 15°; TR 4 s, TE minimum	50 timepoints, ~7 s/phase, contrast injection after 20 s	FOV 24 cm, matrix not specified, slice thickness 5 mm	VFA T1 mapping (FAs 15°, 10°, 5°, 2°)	Software: VersaVue (Invivo) with OmniLook Preprocessing: not specified AIF: not specified Model: extended Tofts two-compartment
8	Vajapeyam S, 2017 [10]	3-T Siemens	Not specified	Gadobutrol, 0.1 mL/kg at injection rate 2 mL/s, the rest not specified	3D T1-weighted fast gradient echo; FA 15°; T4 4 s, TE minimum	50 timepoints, ~7 s/phase, contrast injection after 20 s	FOV 24 cm, matrix not specified, slice thickness 5 mm	VFA T1 mapping (FAs 15°, 10°, 5°, 2°)	Software: VersaVue (Invivo) with OmniLook Preprocessing: not specified AIF: not specified Model: extended Tofts two-compartment
9	Zukotynski KA, 2013 [18]	1.5 T (not specified)	Not specified	Not specified	3D T1-weighted spoiled gradient echo; FA 30°; TR/TE minimum (not specified)	40 dynamics, temporal resolution not specified	FOV 24 cm, matrix ≥128×128, 16 axial slices	No T1/B1 mapping reported	Software: in-house IDL software Preprocessing: not specified AIF: not specified Model: not specified

*3D*, three-dimensional; *AIF*, arterial input function; *FA*, flip angle; *FLASH*, fast low angle shot; *FOV*, field of view;* Gd-BOPTA*, gadobenate dimeglumine;* IV*, intravenous; *MCA*, middle cerebral artery; *ROI*, region of interest; *T*, tesla; *TR*, repetition time; *TE*, echo time; *VFA*, variable flip angle

Studies ranged in cohort size from 6 to 72 patients. Only one study applied the most recent World Health Organization Classification of Tumours of the Central Nervous System (WHO CNS) of 2021 [16, 19]. Three studies investigated histologically confirmed tumours only, while two included some tumours that were histologically confirmed, and four did not mention whether the tumours were histologically evaluated. Four studies investigated untreated tumours, while two dealt with therapy evaluation, and three contained treated and untreated lesions.

The criteria for a meta-analysis were not met. Considerable heterogeneity was observed across cohorts in tumour types, treatment status, and evaluation systems used to diagnose and categorise pathologies. Subsequently, a narrative synthesis was conducted to summarise the key findings of the included studies.

### Risk of bias and imaging standard compliance

Most studies showed a low risk of bias (Table 4), with patient selection description scoring the lowest (Table 4). QIBA compliance, based on the technical parameters presented in Table 3, was limited to low (Table 4), with no study being fully compliant with the recommendations on implementation and reporting.

**Table 4:** Risk of bias analysis of fitting paediatric dynamic contrast-enhanced MRI studies and protocol compliance with quantitative imaging biomarkers alliance (QIBA) recommendations

No.	Author	Risk of bias				Overall score risk of bias	Quantitative imaging biomarkers alliance compliance
		Patient selection	Index test	Reference standard	Flow, timing, and analysis		
1	Arevalo-Perez J, 2024 [17]	Low	Low	Low	Low	Low	Partially compliant
2	Gupta PK, 2017 [15]	Low	Low	Low	Low	Low	Not compliant/insufficiently reported
3	Ho CY, 2025 [16]	Low	Low	Low	Low	Low	Partially compliant
4	Jost SC, 2008 [14]	Medium	Low	Low	Low	Low	Not compliant/insufficiently reported
5	Rochetams BB, 2017 [13]	Low	Low	Low	Low	Low	Not compliant/insufficiently reported
6	Vajapeyam S, 2020 [12]	Low	Low	Low	Low	Low	Partially compliant
7	Vajapeyam S, 2018 [11]	Low	Low	Low	Low	Low	Partially compliant
8	Vajapeyam S, 2017 [10]	Low	Low	Low	Low	Low	Partially compliant
9	Zukotynski KA, 2013 [18]	Medium	High	High	High	High	Not compliant/insufficiently reported

### Noninvasive tumour differentiation

Six studies investigated whether DCE MRI-derived kinetic parameters, including *K*^trans^, *k*_ep_, *v*_e_, and *v*_p_, can differentiate paediatric brain tumour grade or tumour entity [10–13, 15–17]. Where specified, analyses were performed in newly diagnosed, treatment-naïve patients; however, two studies did not report treatment status at the time of imaging and are not included in cumulative analysis.

Four studies investigated DCE parameters in newly diagnosed, treatment-naive patients to differentiate between low- and high-grade paediatric primary brain tumours [10–12, 15, 16]. The cumulative number of cases is approximately 186 after correction for potential cohort overlap (Table 3).

Only Ho et al. (study no. 3, Table 3) applied the most recent WHO CNS 5 classification in their analysis of a mixed primary brain tumour cohort examining the capability of DCE to differentiate low- from high-grade lesions [16]. They present the largest cohort of 36 high-grade (WHO 3 and 4) and 36 low-grade primary brain tumours. No extended Tofts model DCE parameter significantly differed between both groups after Bonferroni correction.

Gupta et al. (study no. 2, Table 3), in their mixed primary paediatric brain tumour study of 64 patients, did not find any significant Bonferroni-corrected differences in extended Tofts model kinetic parameters across different tumour grades, corroborating results by Ho et al. [15]. They did, however, report significantly different (*P*=0.036) *K*^trans^ values between different posterior fossa tumours, namely between pilocytic astrocytomas (0.48, 0.17–1.25.17.25) and medulloblastomas (0.01, 0.0–0.74.0.74). They also observed that ependymomas (8.25 (3.98–17.91)) and pilocytic astrocytomas (8.68 (5–79.05)) had higher *k*_ep_ than medulloblastomas (2.89 (0.0–10.77.0.77); *P*=0.012). Moreover, Gupta et al. reported that medulloblastomas had significantly lower *v*_e_ compared to pilocytic astrocytomas and ependymomas (*P*=0.003 and 0.012, respectively).

Vajapeyam et al. [11] (study no. 7, Table 3) present a comparable mixed histology setup of 41 cases, aiming to differentiate between WHO I and II (LGG by the 2016 WHO CNS classification) and grade III/IV (*n*=25) using extended Tofts model parameters [11]. They conclude that significant differences were observed between groups for *K*^trans^, *k*_ep_ (both higher in HGG), and *v*_e_ (lower in HGG). Statistical error correction was not stated. They had previously published a part of their cohort with a great overlap (study no. 8, Table 3) and concluded that all three parameters had high specificity (range, 82–100%), while the best sensitivity was achieved for *v*_e_ (combined sensitivity 76% vs. 71% for *K*^trans^ and *k*_ep_) [10].

In summary, among definitely treatment-naïve mixed-lesion cohorts, two studies did not identify any differentiation potential for DCE (cumulative cases, *n*=136). In contrast, two studies based on two overlapping datasets, totalling approximately 50 cases, identified diagnostic potential.

Two additional studies evaluated DCE parameters for tumour differentiation or aggressiveness assessment but did not specify treatment status at the time of imaging, limiting interpretability [13, 17]. Rochetams et al. (study no. 5, Table 3) present 10 paediatric patients with mixed gliomas (4 LGG WHO I/II, 6 HGG) of unknown therapy status, with *K*^trans^ ratios being significantly different between grade I and grade IV brain tumours (*P*=0.027), while other differentiations were not possible. Grade IV paediatric brain tumours were distinctly defined by a *K*^trans^ ratio above 0.63 [13]. *K*^trans^-based differentiation between pilocytic astrocytoma and medulloblastoma was impossible. Arevalo-Perez et al. present a 2024 study (study no. 1, Table 3) to differentiate low-grade cerebral ependymoma from anaplastic ones [17]. However, only four cases were anaplastic, and two were low-grade. The *v*_pmax_ was, however, following trends in the adults with higher values in anaplastic lesions (mean 17.44%) than in low-grade lesions (mean 9.65%). Study quality is impaired by very small subgroup sizes and unknown treatment status.

### Treatment response and outcome prediction

Three studies evaluated DCE MRI parameters as markers of treatment response or predictors of clinical outcome in paediatric brain tumours [12, 14, 18].

Zukotynski et al. (study no. 9, Table 3) examined 24 children with mixed primary brain tumour histologies under bevacizumab and irinotecan treatment before, during, and after treatment, with variable data completeness, and only analysed *K*^trans^ (referred to as Kps in the paper). *K*^trans^ did not differ between LGG and HGG at baseline (*n*=21; *P*=0.56). *K*^trans^ was not associated with progression-free survival, but with one exception, lowered during therapy.

Jost et al. (study no. 4, Table 3) present a study of optic pathway LGG patients with unknown therapy status, where they attempted to predict whether a patient would remain clinically stable (*n*=11) or develop aggressive disease (*n*=16) [14]. However, the relation between MRI and clinical judgment is unclear. Nonetheless, they found significantly higher (*P*=0.05) mean *K*^trans^ surface area product (KPS) in clinically aggressive optic pathway gliomas (2.24 mL/min per 100 cm^3^) compared to clinically stable tumours (1.38 ml/min per 100 cm^3^). All clinically stable tumours had a value of <2.0 mL/min per 100 cm^3^. To further support the observed correlation between vascular permeability and aggressiveness, a significantly higher mean permeability value (2.77 mL/min per 100 cm^3^) was observed in sporadic tumours classified as clinically aggressive compared to those classified as clinically stable (<2.0 mL/min per 100 cm^3^) (*P*<0.05).

Finally, the 2020 Vajapeyam et al. publication (study no. 6, Table 3) also contained follow-up scans showing that when analysed as continuous time-dependent variables, associations of mean *v*_e_ with progression-free survival (*P*=0.03) and overall survival (*P*=0.03), as well as maximum *K*^trans^ with progression-free survival (*P*=0.03) were near significant [12]. Greater kinetic parameter increases with time were associated with worse outcomes. Kinetic parameters showed no difference between pseudoprogression and true early progression groups.

## Discussion

### Critical summary

This systematic review on DCE in paediatric brain tumour patients illustrates that only a very few studies have focused on the topic, which is further complicated by substantial heterogeneity in cases and technical execution.

### Clinical findings

A total of nine smaller-scale studies, partially with cohort overlap, unclear or outdated tumour histology, and differing research questions, as well as equivocal scanning parameters, underscore the substantial knowledge gap for DCE applications in paediatric neuro-oncological imaging.

Regarding the differentiation of low-grade and high-grade brain tumours, data relies on four to five studies, with a majority of samples indicating that differentiation is not possible using DCE kinetic parameters. However, the subgroup analyses of these studies suggest that this may be due to the histological heterogeneity of these cohorts, and that a differentiation between two types of tumours may be possible, as indicated by the results of Gupta et al. for pilocytic astrocytoma versus medulloblastoma [15]. Rochetams et al. however, could not find a difference for these two tumour entities, which may be due to their very small sample size of five cases for both groups in total [13]. The equivocal results of the published tumour malignancy prediction studies underscore another issue: it may be easier to gain statistically meaningful results by focusing on two entities in future studies (like medulloblastoma and pilocytic astrocytoma), despite a clinical necessity to be able to differentiate highly malignant from less malignant tumours in a broader set of entities and subtypes. Furthermore, we could not identify a single study that focuses on the molecular differentiation of tumour entities, which, however, is key to modern tumour diagnostics. Approaches, therefore, need to change substantially in the future.

Examining the published nine studies, it is notable that only a minority focused on histologically and clinically comparable cases, such as the optic pathway glioma study by Jost et al. which should be the standard when the sample size is relatively small. In this context, clinical reproducibility is an important factor in advanced MR imaging. While not fitting the inclusion criteria of this publication, the authors want to draw the attention to the publication by Miyazaki et al. who showed in eight paediatric glioma cases that only *K*^trans^ had a variation coefficient below 20% and that the arterial input function (AIF) may show sharper and earlier first pass peaks than in adults having an impact on dynamic scan planning [20]. On the other hand, Carceller et al.’s cohort of five HGG patients showed the lowest variation coefficient for *v*_e_ (2.9%), while also *K*^trans^ and *k*_ep_ remained below 20% [21]. This means that future studies of DCE use in paediatric brain tumour patients must, on the one hand, critically evaluate which parameters to take into account, and, on the other, that AIF selection in the smaller vessels of paediatric brains remains an unsolved but relevant issue. Both are pivotal aspects, particularly in the currently underexplored field of follow-up studies in the paediatric cohort, which leads to the topic of technical implementation in paediatric MRI.

### Technical implementation of dynamic contrast-enhanced imaging in paediatric populations

The reviewed studies illustrate a high heterogeneity in DCE implementation across clinical research settings. Notably, there was a substantial underreporting of key acquisition parameters, particularly with respect to head coil specifications, contrast agent injection parameters, temporal sampling, and post-processing. Scanner field strengths ranged from 1.5 T to 3 T. Head coils in different studies varied from 8 to 20 coil channels, when reported. Contrast administration protocols differed in both type and dosing–most commonly gadobutrol or Gd-BOPTA at 0.1 mmol/kg-although half-dose and repeat-dose strategies were also reported in combination with dynamic susceptibility contrast perfusion MRI, and injection rates varied from 1 mL/s to 5 mL/s. Only a subset of studies incorporated pre-contrast T1 mapping, and B1 mapping was rarely performed. AIF selection varied substantially, from manual middle cerebral artery regions of interest selection to automated extractions, followed by parameter quantification with the extended Tofts two-compartment, Patlak, and leaky tracer kinetic models.

Despite the persistent application of DCE in paediatric neuro-oncology, there are currently no guidelines that specifically address its implementation in children. Existing recommendations, such as QIBA [7], have been developed for adult populations only, and they do not incorporate practical paediatric considerations such as smaller intravenous catheter gauge size [22], the risk of extravasation at higher injection pressures [23], or the need for adjusted dosing strategies [24]. In practice, paediatric patients frequently require the use of 18–21 G catheters, which restrict achievable injection rates. Consequently, lower injection rates are often employed to reduce the risk of extravasation, but slower infusion flattens the AIF peak and compromises the fidelity of tracer kinetic modelling [25]. Reduced AIF sharpness directly limits the accuracy of quantitative parameters such as *K*^trans^, which are sensitive to bolus profile [26]. This gap between existing technical standards and the realities of paediatric practice highlights the need for tailored guidelines that strike a balance between patient safety and the demands of robust pharmacokinetic analysis.

A consistent theme across published studies is the systematic failure to both implement and report acquisitions and analyses in line with QIBA recommendations. Key acquisition parameters, including head coil type, contrast injection details, time resolution, and coverage, were frequently underreported, while essential calibration measures such as T1 and B1 mapping were often omitted. Similarly, descriptions of preprocessing pipelines, AIF definition, and model selection were highly variable or often insufficiently documented, making them difficult to reproduce. The lack of adherence across studies further limits clinical translation and the establishment of robust quantitative biomarkers for paediatric neuro-oncology. Community efforts, such as the Open-Source Initiative for Perfusion Imaging (OSIPI), provide repositories of openly shared software tools and reference data processing pipelines to promote reproducible quantitative DCE imaging [27].

### Limitations of current studies

While DCE demonstrates potentially useful correlations between paediatric brain tumour grade and parameters, including *K*^trans^, *k*_ep_, *v*_e_, and *v*_p_, there are several limitations in the present review that should be considered when interpreting the results.

One important discrepancy in this review is that several included studies varied technically, as described in 4.3, limiting generalisability of the findings across clinical contexts.

Another limitation concerns the substantial variability and discrepancy in tumour type evaluation and grade classification systems. Only four of the nine studies reported compliance with any WHO CNS classification, and of these, only two specified the edition of the WHO CNS classification used. Additionally, several studies did not include histological verification. This may impact the accuracy of the overall conclusions drawn by this review regarding the correlation of DCE parameters with tumour grade and type. For some tumour discriminations, e.g. regarding medulloblastoma vs. pilocytic astrocytoma, the impact of the transition from WHO CNS 2016 to 2021 may be small, and comparisons between such studies can still be made [28], while paediatric glioma classifications underwent substantial adaptations in the 2021 WHO classification [29].

Another crucial limitation identified by this review is the small sample sizes of the studies. This limits the statistical power of the findings and makes it challenging to draw firm conclusions regarding the prognostic value of DCE parameters.

Furthermore, measurement discrepancies may have been introduced by the inconsistent methodologies across studies. Variations in acquisition parameters, including temporal resolution, field strength, contrast agent types, and processing pipelines, are a significant limitation to comparability. Variation in arterial input function (AIF) selection is another relevant issue that may have affected clinical outcomes of the presented studies [30].

The inability to directly compare among studies is exacerbated by the use of different processing models (e.g. Tofts, Patlak, or Kermode models), as each model highlights different aspects of tumour physiology and may not accurately reflect all tumour types. This may pose challenges in producing consistently reliable and accurate results across tumour subtypes.

Lastly, while DCE shows promise in providing insights into tumour vascularity, it possesses some inherent technical limitations. For example, DCE is highly sensitive to motion artefacts, which are particularly evident in paediatric patients who may have trouble staying still during their procedure. Additionally, the use of contrast agents carries a small risk of side effects [8], although these are generally rare.

### Future directions for dynamic contrast-enhanced imaging in paediatric neuro-oncology

Future studies implementing DCE in paediatric neuro-oncology should aim to align more closely with the QIBA recommendations while adapting protocols to the practical constraints of paediatric cohorts. This includes the use of standardised acquisition schemes, a rigorous documentation of contrast injection parameters, and transparent reporting of data processing. Importantly, recommendations specifically tailored to the paediatric population are still lacking, and developing such consensus guidelines will be crucial to strike a balance between technical rigour and the clinical demands of imaging children. In addition, since paediatric tumours are rare and published sample sizes are limited, the creation of inter-institutional databases that compile DCE acquisition and pharmacokinetic parameters would be invaluable, enabling more robust analyses and facilitating clinically translatable biomarkers.

Beyond technical harmonisation, future DCE studies should address biologically and clinically more meaningful questions, moving away from broad comparisons among heterogeneous tumour entities and instead focusing on molecular markers, limited entities, and clinically relevant outcomes to strengthen their translational value. Such outcome analyses entail progression-free and overall survival predictions in specific HGG entities with a dismal prognosis considering aspects of treatment (response), preferably clustering data from several centres to achieve meaningful sample sizes. These steps would foster greater reproducibility, comparability, and clinical impact of DCE in the paediatric neuro-oncology domain, particularly for the benefit of young, highly vulnerable patients.

### Conclusion

DCE MRI may deliver valuable non-invasive imaging biomarkers in paediatric patients with brain tumours, as indicated by several studies. However, the low published sample sizes and technical and case heterogeneity do not currently allow for firm conclusions. Dedicated paediatric DCE MRI protocols, as well as clinically focused studies, are needed. Patients currently do not benefit from clinically used DCE due to still too low levels of evidence for a reliable kinetic parameter interpretation.

## Supplementary Information

Below is the link to the electronic supplementary material.

ESM 1 DOCX (13.5 KB)

## Data Availability

IRB approval was waived for this research. All four authors screened the articles retrieved. In stage 1, only titles and abstracts were screened. In stage 2, full-text articles were screened. An additional hand search was conducted, thoroughly examining references cited in the selected articles to identify additional literature relevant to the research question. A detailed description of the search, retrieval and selection is provided in Supplementary Material S1. QUADAS-2 risk of bias assessments were performed by all authors, with at least two raters evaluating each article [[9]] (https:/paperpile.com/c/ZCQTw0/rezc). Disagreements were resolved by consensus. Compliance with QIBA recommendations for DCE implementation and reporting was assessed for each selected study by an MRI physicist with four years of clinical neuroimaging experience (Y.P.).
