# Supplementary material for: Dynamic contrast-enhanced magnetic resonance imaging in paediatric brain tumours systematically reviewed
Source: Pediatr Radiol. 2026 Apr 1;56(5):1139–54. doi: 10.1007/s00247-026-06600-7 (PMC13134978; doi:10.1007/s00247-026-06600-7)
Supplement: Supplementary file 1 — DOCX (13.5 KB) [file 247_2026_6600_MOESM1_ESM.docx]

**Table 2 Supplement 2: QUADAS-2 categories**

| **Domain 1: Patient selection** | |
| --- | --- |
| **A. Risk of bias** | |
| Signalling questions | RISK: |
| Are the study design, inclusion and exclusion criteria clearly described? | **Low:** Prospective design. The inclusion and exclusion criteria were clearly defined and applied consistently  **Medium:** Retrospective design. All aspects are implicitly findable and defined, but partially left to interpretation  **High:** Study design not clearly stated. The inclusion and exclusion criteria were not clear and have not been applied consistently |
| Was the enrolment sample consecutive or random? | **Low:** Consecutive or random enrolment  **High:** Other or not mentioned sampling method |
| Did the study avoid inappropriate exclusions? | **Low:** No inappropriate exclusions  **High:** Inappropriate exclusions |
| Were the patient's characteristics and demographics (e.g. age, gender, tumour type) adequately described? | **Low:** The patient's characteristics and demographics were adequately described  **Medium:** Yes, minor information details missing or implicit  **High:** The patient's nut characteristics and demographics were missing or not adequately described |
| Was first-line therapy described in case of follow-up studies? | **Low:** In follow-up studies, the first-line therapy is clearly described  **High:** In follow-up studies, the first-line therapy is not clearly described |
| Was the diagnosis confirmed by state-of-the-art methods? | **Low:** The diagnosis was confirmed by state-of-the-art methods  **High:** The diagnosis was not confirmed by state-of-the-art methods |
| RISK: | |
| Could the selection of patients have introduced bias? | **Low** 🡺 0 points  **Medium** 🡺 1 point  **High** 🡺 2 points |
| **Total points** | |
| 0-4 points: **Low risk**  5-8 points: **Medium risk**  9-12 points: **High risk** |  |
| **B. Concerns regarding applicability** | |
| CONCERN: | |
| Is there concern that the included patients do not match the review question? | **Yes/No** |

| **Domain 2: Index test(s)** | |
| --- | --- |
| **A. Risk of bias** | |
| Signalling questions |  |
| Was the Dynamic Contrast Enhanced Magnetic Resonance Imaging (DCE-MRI) technique clearly described and standardized? | **Low:** The DCE-MRI technique was clearly described and standardized  **High:** The DCE-MRI technique was not clearly described and not standardized  **Unclear** |
| Were the index test results interpreted without knowledge of the results of the reference standard? | **Low:** The index results were interpreted in a blinded fashion  **Medium:** There seems to be blinding involved, but the description is implicit  **High:** The index results were (partially) not interpreted in a blinded fashion  **Unclear** |
| Were the criteria for interpreting DCE-MRI results pre-specified and applied consistently? | **Low:** The criteria for interpreting DCE-MRI results were pre-specified and applied consistently  **High:** The criteria for interpreting DCE-MRI results were not pre-specified and not applied consistently  **Unclear** |
| RISK: | |
| Could the conduct or interpretation of the index test have introduced bias? | **Low** 🡺 0 points  **Medium** 🡺 1 point  **High** 🡺 2 points |
| **Total points** | |
| 0-2 points: **Low risk**  3-4 points: **Medium risk**  5-6 points: **High risk** |  |
| **B. Concerns regarding applicability** | |
| CONCERN: | |
| Is there concern that the index test, its conduct, or its interpretation differ from the review question? | **Yes/No** |

| **Domain 3: Reference standard** | |
| --- | --- |
| **A. Risk of bias** | |
| Signalling questions | RISK: |
| Was the reference standard clearly described for differentiation of paediatric brain tumour entities or  tumour progression from therapy-related changes? | **Low:** The reference standard is clearly described  **High:** The reference standard is not described |
| Is the reference standard likely to correctly classify the target condition, e.g. according to up-to-date guidelines? | **Low:** The reference standard is likely to correctly classify the target condition and is in line with common practice  **High:** The reference standard is not based on established guidelines or there is doubt about the classification method |
| Were the reference standard results adequately reported, including any discrepancies or  uncertainties? | **Low:** The reference standard results were adequately reported  **High:** The reference standard results were not adequately reported |
| RISK: | |
| Could the reference standard, its conduct, or its interpretation have introduced bias? | **Low** 🡺 0 points  **Medium** 🡺 1 point  **High** 🡺 2 points |
| **Total points** | |
| 0-2 points: **Low risk**  3-4 points: **Medium risk**  5-6 points: **High risk** |  |
| **B. Concerns regarding applicability** | |
| CONCERN: | |
| Is there concern that the target condition as defined by the reference standard does not match the review question? | **Yes/No** |

| **Domain 4: Flow, timing and analysis** | |
| --- | --- |
| **A. Risk of bias** | |
| Signalling questions | RISK: |
| Was there an appropriate interval between the index test (DCE-MRI) and the reference standard? | **Low:** There is an appropriate interval between index test and reference standard  **Medium:** Unclear interval, but likely appropriate  **High:** There is an inappropriate interval between index test and reference standard |
| Did all patients receive a reference standard and index test? | **Low:** All patients received a reference standard  **High:** Not all patients received a reference standard |
| Did patients receive the same reference standard and index test? | **Low:** All patients received the same reference standard  **High:** Patients did not receive the same reference standard |
| Is the reference standard based on imaging: Are index tests and reference standards performed in a way to allow comparison? | **Low:** Methodologies likely allow for comparison  **High:** Methodologies likely do not allow a comparison |
| Were all patients included in the analysis and were withdrawals explained? | **Low:** All patients were included in the analysis. Withdrawals were explained  **High:** Not all patients were included in the analysis or withdrawals were not explained |
| RISK: | |
| Could the patient flow have introduced bias? | **Low** 🡺 0 points  **Medium** 🡺 1 points  **High** 🡺 2 points |
| **Total points** | |
| 0-3 points: **Low risk**  4-6 points: **Medium risk**  7-10 points: **High risk** |  |

##

## 
